# Effect of using 5A’s model for lifestyle counseling on psychological symptoms in women with polycystic ovary syndrome: a randomized field trial

**DOI:** 10.1038/s41598-022-26274-z

**Published:** 2022-12-17

**Authors:** Fatemeh ZareMobini, Ziba Farajzadegan, Ashraf Kazemi, Mehrdad Salehi

**Affiliations:** 1 Research Center for Nursing & Midwifery Care, Midwifery Department, Nursing & Midwifery Faculty, Shahid Sadogh University of Medical Sciences, Yazd, Iran; 2 Community and Preventive Medicine Department, Medicine Faculty, Isfahan University of Medical Sciences, Isfahan, Iran; 3 Reproductive Health Department, School of Nursing and Midwifery, Isfahan University of Medical Sciences, Hezarjerib Av., Isfahan, Iran; 4 Psychiatry Department, Isfahan University of Medical Sciences, Isfahan, Iran

**Keywords:** Human behaviour, Psychology

## Abstract

Lifestyle modification in women with polycystic ovary syndrome (PCOS) could be associated with increased psychological symptoms. This study aimed to evaluate the effect of lifestyle modification counseling using 5A’s model on the psychological symptoms of women with PCOS. This double-blind, randomized field trial was performed on 70 women with PCOS in two groups of lifestyle modification counseling based on 5A’s model and counseling without using the model. The intervention was performed based on five stages of the 5A’s model (Assess, Advise, Agree, Assist, Arrange) during a week, and psychological symptoms were assessed using Symptom Checklist-90-R before the intervention and one and three months after the intervention. The results showed that one and three months after the intervention, the level of psychological symptoms, except obsessive–compulsive level, were significantly lower in the intervention group than in the control group (*p* < 0.05). Moreover, the level of these symptoms decreased over time in the intervention group (*p* < 0.0001). Using the 5A’s model in lifestyle modification counseling is associated with the promotion of psychological health in women with PCOS, and this model is recommended for lifestyle counseling in women with polycystic ovary syndrome.

## Introduction

Polycystic ovary syndrome (PCOS) is an endocrine disorder in women whose clinical manifestations could be followed by psychological effects such as body image distress^1^, decreased self-esteem^2^, sexual dysfunction^3^, depression, and anxiety^4^. Additionally, the high risk of infertility, diabetes^5^, cardiovascular disease^6^, and breast cancer^7^ endanger the mental health of patients^8^.

Given the metabolic nature of this disorder, lifestyle modification has been accepted as the first line of treatment in women with PCOS^9,10^. Several studies have shown that lifestyle modifications to reduce carbohydrate intake^11^ along with exercise programs can reduce insulin resistance and androgen level and initiate ovulation^12^.

Although a lifestyle modification approach is essential in managing PCOS, following dietary and physical activity recommendations require clients' self-confidence^13^ and self-efficacy^14^. However, the chronic nature of the disease and its manifestations, such as hirsutism, acne, menstrual disorders, infertility^15^, and the social problems caused by it^16^, will reduce patients’ self-confidence^17^ and self-efficacy. Qualitative studies evaluating affected women’s experiences show that this disorder is considered a debilitating disease^18^ with an uncertain future for patients^19^. Nevertheless, obtaining knowledge about the disease is associated with an improved sense of disease control in patients and improves their quality of life and mental health^20^.

Therefore, it is necessary for individuals to receive lifestyle modification counseling based on psychological conditions and needs. Moreover, counseling programs should have the necessary flexibility. In this regard, it is recommended that treatment programs with a multidisciplinary approach be designed and psychological counseling be included in treatment programs for patients with PCOS^10,21^.

Several studies have shown the effect of counseling on the lifestyle of women with PCOS^22,23^. Cognitive-based psychological counseling, such as Cognitive-behavioral therapy, has also resulted in positive outcomes on the mental health of women with PCOS^24^. However, in order to provide lifestyle counseling services for women with PCOS, an integrated model is required^25^. Therefore, counseling can be provided by taking into account the specific psychological conditions of PCOS patients and can promote their mental health.

5A’s is a counseling model that focuses on the individual's cognition of the situation and helps the client to design operational plans for cognition-based lifestyle modification they have received from their living conditions.

5A’s is a behavior change model with five steps including assess, advise, agree, assist, and arrange^26^, and was used in obesity-related counseling for weight loss^26–28^. However, no study has hitherto investigated the effect of lifestyle modification counseling by using this model on clients’ mental health. Accordingly, the present study was conducted to evaluate the effect of a lifestyle modification counseling program based on 5A’s model on the mental health of women with PCOS. Therefore, the aim of the study was to assess the effect of using 5A’s model for lifestyle counseling on psychological symptoms in women with polycystic ovary syndrome.

## Materials and methods

This study is the field trial part of a mixed study conducted in a randomized, double-blind way with the approval of the ethics committee of Isfahan University of Medical Sciences, Iran. The study was conducted on 70 women with polycystic ovary syndrome based on Rotterdam criteria^29^ from August 2019 to January 2020 in Isfahan, Iran. The sample size was calculated for a 1:1 allocation ratio by considering presumed depression score reduction after the intervention, estimated sample attrition, 95% confidence level, and 80% test power with two parallel groups of intervention and control sequentially.

Inclusion criteria included no severe psychological disorders and no movement restrictions. Additionally, women undergoing assisted reproductive treatment were not included in the study. An eligibility assessment was performed by the Ph.D. candidate in reproductive health. Simple random sampling was performed among women referring to the treatment clinics affiliated with Isfahan University of Medical Sciences.

In this study, psychological symptoms were measured on the scales of somatization, interpersonal sensitivity, obsessive–compulsive disorder, depression, anxiety, and hostility using SCL-90-R by one researcher. This checklist was set based on a 5-point Likert scale (0–4) ranging from none (0) to too much (4), and the higher the score, the more psychological symptoms would be^30^. The participants' psychological symptoms scores were measured in both groups before the intervention and one and three months later.

Two midwives, who were familiarized with the process during a session, contributed to the sampling process. For sampling, the files of all patients referred daily to the Gynecology and Endocrinology Clinic were evaluated. Then, an initial interview was conducted with the women with even file numbers, and the eligible ones were invited to participate in the study. After obtaining informed consent and recording the baseline information, SCL-90-R was completed as a self-report. After completing the questionnaires, the participants were introduced to the second midwife for random allocation to the intervention or control groups using 70 cards marked equally as A or B. Each participant took a card out of the envelope identifying one of the research groups. Unlike the first midwife, the participant and the midwife performing the random assignment were unaware of which group each color belonged to. After determining each group, each participant was referred to the first midwife to receive either an intervention or control program.

### Intervention program

An intervention program was developed using 5A’s model in order to provide lifestyle counseling and was performed by one reproductive health specialist. This model included five stages: assess, advise, agree, assist, and arrange, presented individually during four sessions of 45–60 min. With regard to the Assess stage, not only body mass index but also knowledge, beliefs, and lifestyle were assessed in terms of physical activity and nutritional behaviors. In the Advise stage, the disease, the effect of lifestyle on the disease course and symptoms, and behavioral problems of nutrition and physical activity were explained. Computational skills were also explained to balance the received and consumed calories. In the Agree stage, behavioral goals were identified based on each person's interests and priorities, and an agreement was reached with the participant for changing behavior and implementing a practical plan to achieve the goals. These three steps were performed in three sessions.

During the fourth session and regarding the Assist stage, the participant was helped to discuss barriers to the implementation of lifestyle modification practical programs and barrier removal strategies. Moreover, based on the mentioned barriers, each participant was helped to identify the sources of social support related to their problem.

In the Arrange stage, based on the participants' preference, their progress regarding dietary behaviors and physical activity was followed through the telephone call. For the control group, face-to-face nutritional and physical activity recommendations were provided in two sessions with an interval of one week.

Research data were analyzed using SPSS software version 19 and statistical methods of independent t-test, Chi-square, Mann–Whitney, and repeated measure analysis of variance. The significance level in data analysis was lower than 0.05. This field trial was registered at the Iranian Registry of Clinical Trials on 03/10/2017 (IRCT2017092736445N1).

### Ethics approval and consent to participate

All procedures performed on participants were in accordance with the ethical standards of the Isfahan University of Medical Sciences, and informed consent was obtained from all participants.

## Results

**Figure 1:**
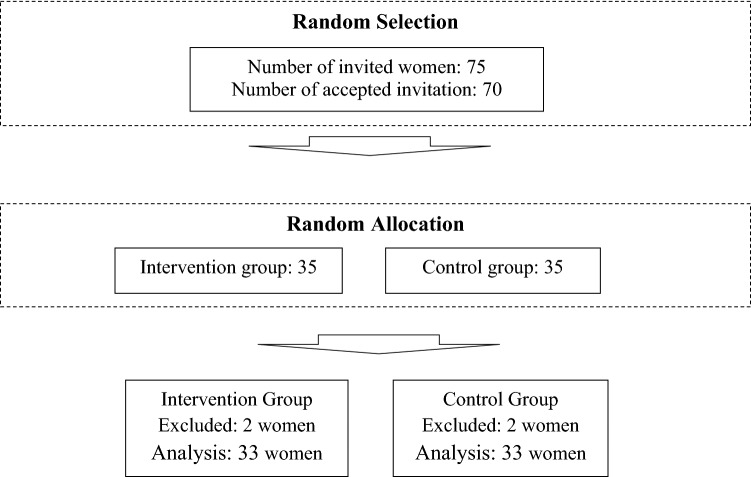
Consort diagram.

Of the 75 eligible women invited to the study, 70 accepted the invitation. One participant in the control group was excluded from the study because of initiating assisted reproductive treatment during the study. Two participants of each group were excluded from the study because of their unwillingness to participate, and finally, 33 subjects remained in each group (Fig. 1). The baseline characteristics of the participants in the two groups are provided in Table 1. Based on the results, the two groups were not significantly different in terms of baseline characteristics (Table 1).

**Table 1:** Comparison of baseline characteristics in study groups.

	Intervention Group (*n* = 33)	Control Group (*n* = 33)	Sig
	Mean (SD) or Number (%)	Mean (SD) or Number (%)	
Age (Year)	29.15 (6.94)	30.15 (6.48)	ns
**Education level (%)**			
Diploma or less	14 (42.40)	20 (60.7)	ns
More than diploma	19 (51.5)	13 (33.3)	
**Occupational status (%)**			
Employed	10 (30.3)	9 (27.3)	ns
Infertility (%)	7 (21.2)	8 (24.2)	ns
Body mass index (kg/m^2^)	28.15 (5.57)	27.21 (4.02)	ns

*SD* standard deviation; *sig* significance; *ns* not significant.

Additionally, the two groups were not significantly different in terms of the measured psychological symptoms before the intervention. There was a significant difference between the mean scores of somatization, interpersonal sensitivity, anxiety, depression, and hostility one and three months after the intervention. The effect of the group on changes in the mean score of somatization, interpersonal sensitivity, anxiety, depression, hostility, and obsessive–compulsive disorders was significant. Accordingly, all psychological symptoms were lower in the intervention group than in the control group one and three months after the intervention (Table 2). According to the results, somatization, interpersonal sensitivity, anxiety, and hostility decreased in the intervention group one and three months after the intervention. The depression likewise decreased after one month; however, its score one month after the intervention was not significantly different from three months after. The level of obsessive–compulsive disorder did not change one and three months after the intervention.

**Table 2:** Comparison of psychologic symptoms and self-efficacy scores between two groups by three times.

	Mean (SD)		Sig^a^	Time/group			
	Int G	Con G		F^b^	Sig^b^	F^c^	Sig^c^
**Somatization**							
At Intake	1.17 (.68)	.80 (.75)	ns				
After 1 month	.55(.53)	.81 (.74)	< .0001	255.73	< .0001	173.20	< .0001
After 3 month	.28 (.38)	1.67 (.57)	< .0001				
**Interpersonal sensitivity**							
At Intake	1.29 (.80)	.96 (.68)	ns	10.65	.001	184.40	
After 1 month	.76 (.55)	1.25 (.68)	.002				< .0001
After 3 month	.38 (.37)	1.54 (.64)	< .0001				
**Obsessive–compulsive disorder**							
At Intake	1.07 (.73)	.88 (.75)	ns	8.57	.002	8.08	.002
After 1 month	1.07 (.74)	.99 (.95)	ns				
After 3 month	1.09 (.72)	.95 (.93)	ns				
**Depression**							
At Intake	1.22 (.76)	.91 (.86)	ns	30.03		63.85	
After 1 month	.48 (.28)	.85 (.88)	.009		< .0001		< .0001
After 3 month	.29 (.32)	.86 (.58)	< .0001				
**Anxiety**							
At Intake	1.05 (.82)	.88 (.67)	ns	7.20	.005	82.40	
After 1 month	.47 (.50)	1.10 (.64)	< .0001				< .0001
After 3 month	.24 (.21)	1.09 (.50)	< .0001				
**Hostility**							
At Intake	.92 (.72)	.81 (.55)	ns	14.67	< .0001	43.36	< .0001
After 1 month	.37 (.41)	.97 (.58)	< .0001				
After 3 month	.23 (.31)	.98 (.58)	< .0001				

*a* Difference between groups; the results of the t test.

*b* test of within subject effect.

*c* test of between subject effect.

*SD* standard deviation; *sig* significance; *ns* not significant.

In the control group, the changes in somatization, interpersonal sensitivity, obsessive–compulsive disorders, and anxiety were significant and upward during the study's three periods. There was no significant difference in the level of depression immediately and one month after the intervention; however, its level increased significantly one month and three months after the intervention. Hostility level also increased in the control group between the first and second measurement times; however, it did not change between one and three months after the intervention (Table 3).

**Table 3:** Pairwise comparison of the psychological symptoms in two groups by three times.

(I): Time	(J): Time	Psychological symptoms											
		Somatization		Interpersonal sensitivity		Obsessive–compulsive disorder		Depression		Anxiety		Hostility	
		(I-J)	Sig	(I-J)	Sig	(I-J)	Sig	(I-J)	Sig	(I-J)	Sig	(I-J)	Sig
**Intervention group**													
At intake	After 1 month	.62	< .0001	.53	< .0001	.000	ns	.74	< .0001	.58	< .0001	.55	< .0001
	After 3 month	.89	< .0001	.91	< .0001	− .003	ns	.93	< .0001	.81	< .0001	.55	< .0001
After 1 month	After 3 month	.27	< .0001	.38	< .0001	.000	ns	.19	.002	.24	< .0001	.14	< .0001
**Control group**													
At intake	After 1 month	− .01	ns	− .29	< .0001	− .11	.002	.06	ns	− .22	.03	− .16	.003
	After 3 month	− .87	< .0001	− .58	< .0001	− .07	ns	.05	ns	− .21	.04	− .17	< .0001
After 1 month	After 3 month	− .86	< .0001	− .29	< .0001	− .04	ns	− .01	ns	.01	ns	− .01	ns

(*I*-*J*) Mean Difference; *sig* significance; *ns* not significant.

## Discussion

The aim of this study was to evaluate the effect of lifestyle modification counseling using 5A’s model on the psychological symptoms of women with PCOS. The results showed that using this model for lifestyle modification counseling could reduce psychological symptoms in these women.

Findings showed that in the intervention group, the levels of somatization, interpersonal sensitivity, anxiety, depression, and hostility symptoms were decreased and were lower than in the control group one and three months after the intervention.

Based on previous studies, using 5A’s model in weight loss counseling resulted in successful lifestyle modification in different groups^26–28^. In 5A’s model, the counselor focuses on the client's knowledge of his or her circumstances and behaviors. In this model, the client is actively involved in designing his/her lifestyle modification program and agrees with the counselor on behavioral goals^28^. In addition, during the following steps, with the help of a counselor, planning is done to improve the lifestyle.

The positive effect of client participation in nutrition counseling for chronic patients has been previously reported on counselor-client relationships^31^, and this approach has been accepted as a critical part of evidence-based practice^32^. Moreover, based on the results of a study, it is necessary to include unique psychological considerations of the disease in the behavioral interventions for the lifestyle of women with PCOS to encourage them to participate in such studies^10^.

The clients' participation in the 5A’s-based counseling program provides an opportunity for them to plan for lifestyle modification based on their specific psychological conditions and limitations and not to become anxious after receiving lifestyle modification counseling. It was confirmed by another research finding indicating an increase in psychological symptoms in the control group.

These results suggest that although lifestyle counseling for the modification of dietary behaviors and physical activity without considering the psychological condition of PCOS patients may lead to lifestyle improvements, it increases psychological symptoms as well. These results are in line with Hassan et al., who reported that anxiety and depression disorders were related to an unhealthy lifestyle in women with PCOS^33^.

These results show that women with PCOS need nutritional advice, physical activity, psychological support, and increased self-efficacy to control the condition. According to Lin et al., most women who were aware of the complications of PCOS believed that some critical complications of the disease could not be controlled by diet modification and physical activity^34^. However, during 5A’s model, the counselor is provided with an opportunity to correct the misconceptions by identifying these factors. Thus, this research confirmed that using these opportunities can improve the mental health of the participants with PCOS. Assessing experiences in women with PCOS has shown that these women need to be psychologically supported to modify their lifestyle^35^. According to another study, implementing supportive programs has effectively empowered women with PCOS to manage their lifestyle^36^.

The client's participation in developing a lifestyle modification program during multiple stages of designing a counseling model based on 5A’s model is another feature of this model. Using this model, the client gains the necessary knowledge not only about the disease and the impact of behavior change programs but also about planning for lifestyle modification. The client’s participation in planning can lead to self-confidence in controlling the situation^32,35^. Additionally, the patient’s perception of the controllability of the disease can be associated with disease acceptance^14^ and subsequent mental health promotion.

According to another study finding, unlike the intervention group, the level of obsessive–compulsive disorder in the control group increased during the study.

This finding shows that using this model is not associated with decreased obsessive–compulsive disorders; however, the increase in this symptom in the control group in the one- and three-month follow-up indicates that counseling can prevent the increase in obsessive–compulsive disorder in the recipients of lifestyle modification counseling.

The high risk of obsessive–compulsive disorder in women with PCOS has been reported in previous studies^37^. The present study showed that lifestyle modification counseling in women with PCOS without considering their psychological condition might increase their psychological symptoms.

The present study did not evaluate changes in lifestyle-related behaviors. The results, thus, have to be interpreted by considering this limitation because the difference in following the recommendations of lifestyle modification in the two groups may be related to psychological changes, which could not be examined in this study and is recommended to be considered in future studies. Moreover, due to the financial limitations of the research project, the effect of the intervention on the women's mental health was only followed up to three months, which was another limitation of the study.

This study has provided a client-centered model for lifestyle modification counseling in women with PCOS, which can also moderate their psychological burden. The results indicated that using 5A’s model for lifestyle modification counseling can lead to positive outcomes concerning the psychological health of women. Therefore, this model is recommended for providing lifestyle modification counseling programs in infertility centers and centers providing health services.

## Data Availability

Data and material are available on request from the corresponding author.
